# Enhancing Nutritional Quality and Functionality of Legumes: Application of Solid‐State Fermentation With *Pleurotus ostreatus*

**DOI:** 10.1002/fsn3.70783

**Published:** 2025-10-25

**Authors:** Seyyed Mohammad Ali Noori, Mohammad Hojjati, Reihaneh Sorourian

**Affiliations:** ^1^ Toxicology Research Center, Medical Basic Sciences Research Institute Ahvaz Jundishapur University of Medical Sciences Ahvaz Iran; ^2^ Department of Nutrition, School of Allied Medical Sciences Ahvaz Jundishapur University of Medical Sciences Ahvaz Iran; ^3^ Department of Food Science and Technology Agricultural Sciences and Natural Resources University of Khuzestan Mollasani Iran; ^4^ Department of Food Science and Technology, North Tehran Branch Islamic Azad University Tehran Iran

**Keywords:** anti‐nutritional factors, legumes, protein digestibility, solid‐state fermentation

## Abstract

Legumes are recognized for their nutritional and economic value as a high‐quality protein source. However, antinutrients present in legumes can hinder digestibility and nutrient absorption, ultimately limiting the bioavailability of essential dietary components. Various processing techniques, including enzymatic hydrolysis, plant breeding for reduced antinutrient content, and traditional methods like soaking, germination, cooking (boiling and roasting), can effectively mitigate antinutrient levels and enhance protein digestibility in legumes. Solid‐state fermentation (SSF) presents a bioprocessing approach that utilizes microbial communities, including both macro and micro‐organisms, to degrade antinutrients and enhance the nutritional profile of legumes. This study aimed to process lentils, chickpeas, and soybeans by SSF with the fungus Pleurotus ostreatus. The effect of SSF on the nutrient and anti‐nutrient components of lentils, chickpeas, and soybeans was evaluated. The results of this work illustrated that lentils, chickpeas, and soybeans processed by SSF with *P. ostreatus* increased the protein contents. Lower contents of phytate and tannin of fermented lentils, chickpeas, and soybeans were achieved by SSF. In addition, SSF with *P. ostreatus* significantly increased the inhibitory effects against DPPH radicals in lentils, chickpeas, and soybeans. Fermentation also increased the digestibility of proteins in the investigated legumes. Based on the results, lentils, chickpeas, and soybeans processed by SSF with *P. ostreatus* can be introduced in the development of new functional foods or as a new food ingredient used in food design. In conclusion, solid‐state fermentation (SSF) enhances the nutritional quality of legumes by increasing protein digestibility, improving antioxidant potential, and reducing antinutrients. This process not only makes legumes more nutritionally valuable but also contributes to better health outcomes by improving the bioavailability of essential nutrients.

## Graphical abstract

This study aimed to process lentils, chickpeas, and soybeans by SSF with the fungus Pleurotus ostreatus. The effect of SSF on the nutrient and anti‐nutrient components of lentils, chickpeas, and soybeans was evaluated. Based on the results, lentils, chickpeas, and soybeans processed by SSF with *P. ostreatus* can be introduced in the development of new functional foods or as a new food ingredient used in food design.

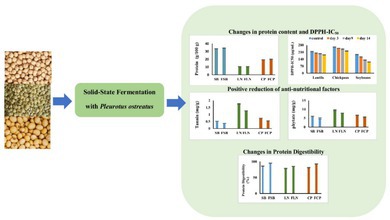

## 1 Introduction

Based on the projections of the United Nations, the global population will increase to 9.7 billion in 2050 (United Nations 2015). This demographic expansion will naturally enhance the requirement for food (Henchion et al. 2017). Therefore, providing enough protein and other nutrients to the growing population requires more sustainable alternatives to animal foods (Lappi et al. 2022). In this regard, the application of plant protein sources (such as legumes and grains) has received much attention due to their environmental sustainability (Heredia and Andr 2021). According to reports, legume cultivation has a high economic and environmental advantage due to its capability to fix nitrogen to replace synthetic fertilizers and thus decrease emissions of greenhouse gases (Khazaei et al. 2019). In general, legumes are of high importance in food security and sustainable agriculture worldwide. Legumes are the second most important food after cereals in terms of the level of human consumption (Dhull et al. 2020; Samtiya et al. 2020). Legumes are a good source of protein, fiber, carbohydrates, and contain low fat and low glycemic index (Rahate et al. 2021). Dietary antioxidants such as legume proteins in the diet lead to protective effects for several chronic diseases such as hypercholesterolemia, type 2 diabetes, cardiovascular diseases, and cancer (Rahate et al. 2021). The quality of proteins in food is determined by the Biological Value (BV) index, which measures the proportion of absorbed protein utilized by the body. The BV is influenced by the amino acid composition of the food (Rahate et al. 2021). Studies indicate that animal proteins generally have a higher BV compared to plant proteins. For instance, the BV of faba beans and soybeans ranges from 40 to 48 and 58 to 69, respectively, which is significantly lower than that of eggs (97) and milk (91) (Rahate et al. 2021). On the other hand, anti‐nutritional factors such as trypsins, saponins, phytic acid, tannins, and lectins have a negative effect on the biological value of protein (Rahate et al. 2021; Revilla 2015). In general, although legumes are a rich source of macronutrients and micronutrients, they have limited bioavailability and digestibility due to anti‐nutritional factors (Heredia and Andr 2021; Nkhata et al. 2018). The anti‐nutritional compounds of legumes lead to irritable bowel syndrome and food allergies (Gänzle 2020). Therefore, investigating various treatments (such as pressure cooking, boiling, soaking, and germination) to eliminate anti‐nutritional factors is of great importance (Rahate et al. 2021). In this regard, biological methods such as enzymatic treatments, germination, and fermentation were stated to be more effective (De Pasquale et al. 2021).

Fermentation has been proposed as an effective strategy to enhance the tolerance of plant‐based foods for sensitive individuals (Gänzle 2020). Beyond improving rheological and sensory properties and reducing or eliminating anti‐nutritional factors, fermentation is considered the most suitable approach for unlocking the full nutritional and functional potential of vegetable matrices and cereal‐based foods (De Pasquale et al. 2021). Fermentation is typically carried out through two main methods: submerged fermentation (SmF) and solid‐state fermentation (SSF), both of which rely on microorganisms and their enzymatic activities (Nkhata et al. 2018). Studies suggest that SSF is particularly well‐suited for fungal growth, as fungal hyphae can effectively colonize the spaces within solid particulate substrates. However, the low moisture content in SSF can limit biological activity. In contrast, SmF is more favorable for bacterial cultivation due to bacteria's requirement for higher water activity (Garrido‐Galand et al. 2021). Research highlights SSF as an environmentally friendly option, offering advantages such as higher volumetric efficiency, greater product concentration, reduced water activity, and simpler, more cost‐effective downstream processing compared to SmF (Garrido‐Galand et al. 2021). Additional benefits of fermentation include low effluent generation, non‐toxicity, ease of cultivation, and cost‐effectiveness (Dhull et al. 2020; Xiao et al. 2021).

During SSF, enzymes such as amylases, lipases, and proteases may be produced by microorganisms that hydrolyze carbohydrates, lipids, and proteins into more digestible compounds with a more pleasant taste and aroma (Dhull et al. 2020). Microorganisms, especially fungi, have been reported to secrete numerous enzymes capable of hydrolyzing glycosidic, ester, or ether bonds, leading to the breakdown of cell wall structural components. As a result, during solid‐state fermentation (SSF), insoluble phenolic compounds covalently bound to the cell wall are released, enhancing the biological activities of fermented legume products (Xiao et al. 2021). Consequently, SSF can be utilized as a promising food processing technology to improve the biological activities, phytochemical composition, and nutritional value of legume‐based products. Given these benefits, *P. ostreatus* is considered an excellent microbial candidate with significant potential for producing value‐added fermented foods.

However, the fermentation process has been mostly done by yeast and bacteria, and macrofungi have received less consideration for solid‐state fermentation. Therefore, this research project aims to determine the efficacy of solid‐state fermentation by *Pleurotus ostreatus* on the nutrient and anti‐nutrient content and antioxidant profiles of lentils, chickpeas, and soybeans.

## 2 Materials and Methods

### 2.1 Materials

The chickpeas ( *Cicer arietinum* ), lentils ( *Lens culinaris* ), and soybeans ( *Glycine max* ) were obtained from the local food market in Ahvaz, Iran. The *Pleurotus ostreatus* CS155 was obtained from a mushroom production center (Karaj, Iran). Agar powder, mycopeptone, malt extract, and sodium carbonate (Na_2_CO_3_) were obtained from Scharlab (Barcelona, Spain). Glucose (C6H12O6), 2,2‐diphenyl‐1‐picrylhydrazyl (DPPH), methanol (CH3OH), dimethylsulfoxide (DMSO), sodium hydroxide (NaOH), and trichloroacetic acid (TCA) were acquired from Sigma‐Aldrich (St. Louis, MO, USA). Hydrochloric acid (HCl) was purchased from AppliChem Panreac (USA). All other reagents and chemicals applied in the present study were of analytical reagent grade.

### 2.2 Fungal Solid‐State Fermentation (SSF)

To preparation of starter culture, *P. ostreatus* was isolated from the agar plate and then cultured in Petri dishes containing agar (1.5%), glucose (2%), mycopeptone (0.1%) and malt extract (2%) and incubated at 28°C for 14 days. After this time, the obtained mycelium was again inoculated in the culture broth containing malt extract (2%), glucose (2%), and mycopeptone (0.1%), and incubated for 14 days at 28°C. Finally, the resulting broth was applied as a starter culture for the fermentation of legumes (Sánchez‐García et al. 2022).

Legume seeds were washed with deionized water, and their humidity was adjusted to about 65% (distilled water ratio of 1:0.65 (w/v) was used for seeds). Then, the legumes were sterilized at 121°C for 20 min. *P. ostreatus* suspension was then inoculated in the glass jars containing sterilized lentils, chickpeas, and soybean substrates (5 mL/100 g wet seed). The glass jars were kept at 28°C in a microbial incubator under static conditions. Non‐fermented lentils, chickpeas, and soybeans without *P. ostreatus* suspension inoculation were also applied as a control. After the fermentation time, the samples were freeze‐dried and crushed by a grinder, and the resulting flours were sieved (60 mesh sieve) and kept in the refrigerator until analysis (Sánchez‐García et al. 2022; Xiao et al. 2021).

### 2.3 Proximal Chemical Analysis

The Association of Official Analytical Chemistry methods were used to analyze proximal parameters (Association of Analytical Communities (AOAC) 2006). Kjeldahl method (AOAC 930.29), Goldfish method (AOAC 920.36C), gravimetric method (AOAC 14.006) and moisture method (AOAC 930.15) were used to evaluate the content of protein, fat, ash, and moisture of fermented and non‐fermented lentils, chickpeas, and soybeans. Carbohydrates were determined by difference (Rengadu et al. 2020).

### 2.4 pH

The pH of the sample slurry (mixed 1.5 g of the seeds with 10 mL of distilled water) was determined by a pH meter (ADWA Bench Meter, ad8000) (Rani et al. 2018).

### 2.5 Total Titratable Acidity

To determine total titratable acidity (TTA), a few drops of phenolphthalein indicator were added to a homogeneous solution of the sample (3 g) and distilled water (20 mL). Then the resulting solution was titrated by NaOH (0.1 N) until reaching the endpoint; (Rani et al. 2018):

$$\text{Lactic acid}\%=\frac{\text{Volume of NaOH consumed}\times 0.1\times \text{final volume}\times \text{equivalent weight of acid}\times 100}{\text{weight of sample taken}\times \text{Volume of sample taken}\times 1000}$$

### 2.6 Antioxidant Activity

The antioxidant potential of fermented and non‐fermented legume seeds was assessed using DPPH free radical scavenging activity according to previous methods with some modifications (Sharma and Giri 2022). The legume extract solutions (0.1–0.9 mg/mL) were prepared in 50% DMSO. Then, DPPH solution was added to each solution, and the resulting mixture was incubated for 30 min in the dark at 37°C. The absorbance of the reaction solution was recorded at 517 nm with a UV–Vis spectrophotometer. The percentage of DPPH scavenging rate was determined using the following equation (Sorourian et al. 2022):

$$\text{DPPH scavenging rate}\;\left(\%\right)=\frac{\left(\text{Ablank}-\text{Asample}\right)}{\text{Ablank}}\times 10$$

where As and Ab are the absorbances of sample solutions and the control reaction (without the sample), respectively.

### 2.7 Anti‐Nutritional Factors

A rapid colorimetric procedure was used to determine the phytate content of fermented and non‐fermented legumes (Xu et al. 2016). Briefly, samples were extracted with aqueous HCl (2.4%) for 1 h, and the resulting slurry was centrifuged (4000 ***g*** for 10 min). Inorganic phosphorus and other interfering compounds present in the diluted supernatant were removed by passing through AG1‐X8 anion exchange resin. Phytate was eluted by applying NaCl (0.7 M). Eventually, its concentration was evaluated based on the reaction among ferric ions and sulfosalicylic acid in Wade's reagent by spectrophotometer at 500 nm.

AOAC 952.03 method was used to determine the tannin content of fermented and non‐fermented legumes (Association of Analytical Communities (AOAC) 2006; Espinosa‐Páez et al. 2017). Briefly, 100–800 aliquots in 1000 μL of a tannic acid stock solution of 0.1 mg/mL were applied to prepare a standard curve. Then, the solution (1 mL) obtained by dissolving the sample (10 mg) in water (10 mL) was taken for determination. Then a mixture of water (7.5 mL), Folin–Denis (500 μL) and Na_2_CO_3_ (1 mL, 35%) was prepared and kept for 30 min at room temperature. The reaction solution's absorbance was recorded at 760 nm by spectrophotometer; eventually, the results were presented as tannic acid equivalent.

### 2.8 In Vitro Protein Digestibility

In vitro protein digestibility of fermented and non‐fermented legumes evaluated based on previous studies with a slight modification (Espinosa Páez et al. 2023). In the first step, the simulated digestion protocol was prepared based on using digestive enzymes with simulated liquids. For this purpose, the sample (5 g) was placed in a 50 mL tube. The oral phase was prepared by adding simulated oral fluid (5 mL, SOF) and incubating at 37°C for 5 min with agitation. To prepare the gastric phase, simulated gastric fluid (12 mL, SGF) was mixed with 3850 U/mg gastric porcine pepsin at pH 2.3 and incubated at 37°C for 2 h at 55 rpm with orbital agitation. The final phase was prepared by mixing simulated intestinal fluid (20 mL, SIF), pancreatin porcine pancreas 8 USP (1.98 mg), and bile bovine pancreas at pH 8 and incubating at 37°C for 2 h under orbital agitation. Then, the protein digestibility of the samples was determined in the products of simulated digestion. For this purpose, an intestinal phase product (37 mL) was obtained. After precipitating the undigested protein with an aqueous solution of trichloroacetic acid (12%, w/w), the sample was centrifuged (3500 rpm for 15 min). Finally, the protein digestibility of fermented and non‐fermented legumes was determined based on the extracted protein content after digestion and the content of the total soluble protein.

### 2.9 Statistical Analysis

Data statistical analyses were carried out with the IBM SPSS Statistics 23 program (SPSS Inc., Chicago, IL, USA) using one‐way analysis of variance (ANOVA) and Duncan's test multi‐range tests (*p* < 0.05).

## 3 Results and Discussion

### 3.1 Proximal Chemical Analysis

The proximate compound refers to the main ingredients of the food such as protein, fat, ash, moisture, and carbohydrates. The results of the chemical composition of non‐fermented and fermented lentils, chickpeas, and soybeans are presented in Table 1. As can be seen, fermentation has led to an increase in the protein content of the studied legumes, which can be attributed to the synthesis of amino acids as a result of fermentation (Espinosa‐Páez et al. 2017). In the study of Espinosa‐Páez et al., fermentation of oats and kidney beans by *P. ostreatus* increased protein content, which was in agreement with the results of the present study (Espinosa‐Páez et al. 2017). The increment in protein may also be attributed to the enhancement in unicellular protein biomass resulting from *P. ostreatus* growth (Deepalakshmi and Mirunalini 2014). Also, according to reports, *P. ostreatus* has a tannase effect, so reducing the tannin content leads to the reduction of tannin‐protein complexes, thus increasing protein availability (Espinosa‐Páez et al. 2017). Fermentation led to decreased fat in lentils, chickpeas, and soybeans, which can be attributed to the breakdown of lipids with lipase and their usage as a source of energy for bacteria (Knez et al. 2023). Similarly, in the study of Espinosa‐Páez et al., fermentation of kidney beans and black beans by *P. ostreatus* reduced fat content (Espinosa‐Páez et al. 2017). However, the fermentation of oats increased fat content. According to reports, oats, rice, corn, wheat, and grains of beer are more accessible substrates to the fungus, so increased fat content in oats substrates can be attributed to the greater growth of the *P. ostreatus in these* substrates and the fat contained in the *P. ostreatus* (Espinosa‐Páez et al. 2017). The increase in protein and ash percentage can be attributed to the increase in bacterial biomass and the loss of carbohydrates due to fermentation (Emkani et al. 2022).

**TABLE 1:** Nutritional components of various obtained flours.

Parameters	SB	FSB	LN	FLN	CP	FCP
Protein	33.32 ± 0.19	34.35 ± 0.38	10.33 ± 0.24	10.62 ± 0.17	19.31 ± 0.26	20.03 ± 0.23
Ash	4.27 ± 0.07	4.01 ± 0.07	2.33 ± 0.09	2.35 ± 0.06	3.51 ± 0.24	3.51 ± 0.10
Fat	19.15 ± 0.10	19.06 ± 0.10	0.41 ± 0.041	0.40 ± 0.01	5.45 ± 0.23	5.15 ± 0.20
Moisture	13.59 ± 0.11	14.12 ± 0.15	14.12 ± 0.11	14.61 ± 0.18	14.81 ± 0.16	15.21 ± 0.40
Carbohydrates	29.67 ± 0.34	28.46 ± 0.24	72.81 ± 0.12	72.02 ± 0.23	56.92 ± 0.17	56.10 ± 0.34

*Note:* Results are represented in g/100 g dry basis for proximate composition.

Abbreviations: CP, chickpeas; FCP, fermented chickpeas; FLN, fermented lentils; FSB, fermented soybeans; LN, lentils; SB, Soybeans.

### 3.2 pH and Total Titratable Acidity (TTA)

The initial pH of lentils, chickpeas, and soybeans was 6.64, 6.74, and 6.50, respectively, and changed to 5.46, 5.25, and 15.31 after fermentation. As can be seen (Table 2), fermentation led to a significant decrease (*p* < 0.05) in the pH of lentils, chickpeas, and soybeans. TTA of lentils, chickpeas, and soybeans increased significantly due to fermentation (*p* < 0.05). During fermentation, the activity of organisms breaks down carbohydrates into organic acids. Therefore, the decrease in pH and increase in TTA due to fermentation can be attributed to the increase in organic acids produced by organisms during fermentation (Olukomaiya et al. 2020). The results of the evaluation of fermentation efficiency on TTA and pH were very diverse in different studies because the formation of organic acids is influenced by the strain used for fermentation (Bartkiene et al. 2015). It has been stated that the TTA of lupin flour increased due to SSF, which was in line with the results of the present study (Olukomaiya et al. 2020). It has also been stated that the pH of black chickpea flour decreased significantly (*p* < 0.05) during fermentation (De Pasquale et al. 2021). In another study, it was reported that the pH of the media increased in the first 36 h of fermentation, and after this time, the pH changes were not significant (*p* > 0.05) (Chutmanop et al. 2008).

**TABLE 2:** pH and TTA of different obtained flours.

Parameters	pH	Total titratable acidity (g lactic acid/g sample)
SB	6.50 ± 0.00^c^	0.75 ± 0.00^d^
FSB	5.31 ± 0.01^e^	1.78 ± 0.01^b^
LN	6.64 ± 0.00^b^	0.62 ± 0.01^e^
FLN	5.46 ± 0.01^d^	1.65 ± 0.00^c^
CP	6.74 ± 0.00^a^	0.53 ± 0.00^f^
FCP	5.25 ± 0.01^f^	1.84 ± 0.01^a^

*Note:* Different lowercase letters indicate a significant difference (p < 0.05) in various obtained flours.

Abbreviations: CP, chickpeas; FCP, fermented chickpeas; FLN, fermented lentils; FSB, fermented soybeans; LN, lentils; SB, Soybeans.

### 3.3 Antioxidant Activity

**FIGURE 1:**
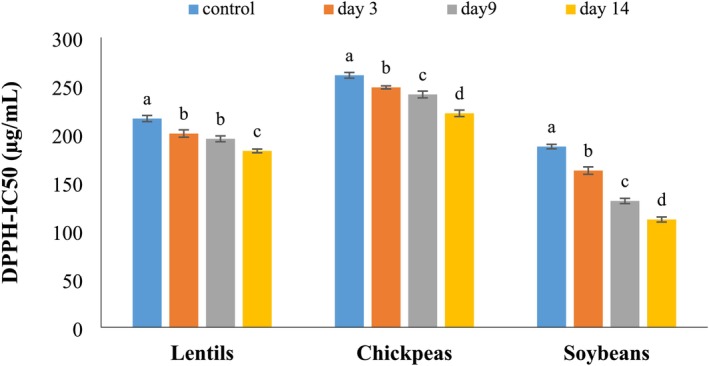
Efficacy of solid‐state fermentation by *P. ostreatus* on the DPPH radical scavenging potential. Significant differences (*p* < 0.05) between the various fermentation times of each sample were shown with lowercase letters.

Fermentation of grains and legumes produces compounds with higher antioxidant capacity. In extensive studies, the potential of fermentation to increase the total polyphenolic content and antioxidant activities of the cereal or legume has been reported (Dulf et al. 2016; Xiao et al. 2021). This study evaluated the DPPH radical scavenging activity of lentils, chickpeas, and soybeans on the third, ninth, and fourteenth days of fermentation, with the results presented in Figure 1. Non‐fermented samples' DPPH radical scavenging activity was also examined as a control. As can be seen, the IC_50_ of fermented samples was significantly (*p* < 0.05) lower than in control samples, indicating that fermentation increased the DPPH radical scavenging potential. The DPPH‐IC_50_ of lentils, chickpeas, and soybeans on the third day of fermentation was 200.47, 248.24, and 162.15 μg/mL, respectively. These values decreased to 195.04, 240.92, and 130.72 μg/mL, respectively, on the ninth day. The lowest DPPH‐IC50 was also observed on the 14th day of fermentation, which were 182.50, 221.44, and 111.53 μg/mL for lentils, chickpeas, and soybeans, respectively. Since lower DPPH‐IC50 indicates better antioxidant activity, it can be stated that the antioxidant activity of the samples was dependent on fermentation time and increasing fermentation time led to increased antioxidant activity.

According to Pasquale et al. study, it was reported that antioxidant activities of black chickpea flour increased with fermentation processing by *L. plantarum* (De Pasquale et al. 2021). Espinosa‐Páez et al. proved that the antioxidant capability of black soybeans increased due to SSF with *Eurotium cristatum* YL‐1 (Xiao et al. 2021). It has also been reported that the antioxidant capability of soybeans increased during fermentation by *Eurotium cristatum* (Chen et al. 2020). According to reports, the increment in antioxidant potential of fermented samples can be related to the phenolic compounds released during the SSF process (Chen et al. 2020). According to reports, fermentation causes biological alteration in the substrate, which leads to the production of biologically active secondary metabolites and thus enhances their antioxidant activity (Dhull et al. 2020). Fungi release aglycones through the production of hydrolytic enzymes during fermentation, which increases the antioxidant activity by increasing the content of phenolic and anthocyanin compounds (Dhull et al. 2020; Heredia and Andr 2021).

### 3.4 Anti‐Nutritional Factors

Legumes contain some components (tannins, phytic acid, saponins, galactosides, isoflavones, trypsin, and biogenic amines), classified as anti‐nutritional factors, that negatively affect the nutritional quality of legume ingredients (Emkani et al. 2022). The galactosides of sucrose cause digestive discomfort and flatulence due to fermentation by gut bacteria in the large intestine (Emkani et al. 2022). Phytic acid also forms a complex with iron, copper, magnesium, calcium, and manganese and thus reduces their absorption in the gastrointestinal tract (Emkani et al. 2022). (Arbab Sakandar et al. 2023). During fermentation, several enzyme activities (decarboxylase, polyphenol oxidase, phytases and tannase) lead to the degradation of anti‐nutritional compounds (De Pasquale et al. 2021). Data in Table 3 present the effect of fermentation on tannins and phytate contents in lentils, chickpeas, and soybeans. Lentils have higher tannin and phytate content than other studied legumes. Tannin contents in lentils, chickpeas, and soybeans were 1.76, 0.74, and 0.52 mg GAE/g sample, which after fermentation decreased to 1.27, 0.55, and 0.37 mg GAE/g sample, respectively. A significant reduction was observed in the phytate content of legumes due to fermentation. Due to fermentation, the phytate content of lentils, chickpeas, and soybeans decreased from 9.55 to 7.93, 6.67 to 5.60, and 6.06 to 4.92 mg/g samples. There are similarities between the results obtained from anti‐nutritional factors in this study and those described by Espinosa‐Páez, who observed tannin reduction from 65.21 to 22.07 mg/100 g and 55.67 to 28.11 mg/100 g in black beans and oats as a result of fermentation (Espinosa‐Páez et al. 2017). According to Pasquale et al.'s study, the content of black chickpea flour's anti‐nutritional compounds (tannins, raffinose and saponins) decreased due to fermentation with *L. plantarum* (De Pasquale et al. 2021). Microorganisms used in the fermentation of legumes can reduce the level of anti‐nutritional factors by changing their chemical composition. According to reports, the function of tannase in fungi leads to the reduction or elimination of tannins (Espinosa‐Páez et al. 2017).

**TABLE 3:** Protein digestibility and anti‐nutritional factors of different obtained flours.

	Protein digestibility (%)	Anti‐nutritional factors (mg/g)	
		Tannin content	Phytate content
SB	85.84 ± 0.94^c^	0.52 ± 0.00^d^	6.06 ± 0.21^d^
FSB	95.15 ± 0.57^a^	0.37 ± 0.00^e^	4.92 ± 0.38^f^
LN	78.63 ± 1.07^e^	1.76 ± 0.03^a^	9.55 ± 0.28^a^
FLN	85.02 ± 0.52^c^	1.27 ± 0.01^b^	7.93 ± 0.03^b^
CP	81.09 ± 1.02^d^	0.74 ± 0.00^c^	6.67 ± 0.16^c^
FCP	92.63 ± 0.74^b^	0.55 ± 0.01^d^	5.60 ± 0.16^e^

*Note:* Different lowercase letters indicate a significant difference (p < 0.05) in various obtained flours.

Abbreviations: CP, chickpeas; FCP, fermented chickpeas; FLN, fermented lentils; FSB, fermented soybeans; LN, lentils; SB, Soybeans.

### 3.5 In Vitro Protein Digestibility

Recently, the development of vegetable protein sources has attracted much attention. However, plant protein sources often have lower biological quality than animal protein sources (Espinosa Páez et al. 2023). Protein digestibility is defined as how humans hydrolyze protein (Emkani et al. 2022). Table 3 shows that fermented lentils, chickpeas, and soybeans have higher protein digestibility than non‐fermented legumes. The protein digestibility of non‐fermented lentils, chickpeas, and soybeans was 78.63%, 81.09%, and 85.84%; which increased to 85.02%, 92.63%, and 95.15% after fermentation. This study's findings mirror previous studies investigating the effect of fermentation by *P. ostreatus*. *Pleurotus ostreatus* has a high selectivity of delignification, which causes substrate degradation and increases protein digestibility (Espinosa‐Páez et al. 2017). In addition, the fungus's ability to decrease anti‐nutritional compounds leads to increased protein digestibility (Espinosa‐Páez et al. 2017). Pasquale et al. proved that the protein digestibility of black chickpea flour increased after fermentation with *L. plantarum* (De Pasquale et al. 2021). Similarly, it was stated in Espinosa‐Páez et al.'s study that solid‐state fermentation by *P. ostreatus* led to increased protein digestibility of black beans, kidney beans, and oats (Espinosa‐Páez et al. 2017). Legume protein digestibility is increased by proteolytic and tannase activities in the organisms used in fermentation (Heredia and Andr 2021). Since the formation of complexes between tannins/phytates and proteins affects protein digestibility and bioavailability, the reduction of phytate and tannin content can be considered a factor in increasing protein digestibility (Heredia and Andr 2021; Shrivastava and Chakraborty 2018). On the other hand, proteolytic activities lead to protein hydrolysis into small peptides and amino acids, which increases protein digestibility (Heredia and Andr 2021). The decrease of anti‐nutritional compounds due to fermentation limits protein cross‐linking and makes proteins susceptible to proteolytic attacks (Emkani et al. 2022).

## 4 Conclusions

In this study, SSF with the fungus *P. ostreatus* significantly improved the nutritional value of fermented lentils, chickpeas, and soybeans. Based on the results, SSF led to an increase in protein digestibility and antioxidant activity during fermentation. Fermentation also reduced the content of tannin and phytate in the investigated legumes. Therefore, fermented lentils, chickpeas, and soybeans can be introduced as suitable compositions to develop new functional foods or as an alternative to animal‐origin protein. In addition, it is essential to investigate the impact of fermented lentils, chickpeas, and soybeans on their potential beneficial health effects with in vivo studies for the production of functional foods. SSF with the fungus *P. ostreatus* was considered an efficient bioprocess for enhancing the biological activity of lentils, chickpeas, and soybeans. However, the potential and benefits of solid‐state fermentation (SSF) must be evaluated on a case‐by‐case basis, as certain limitations, such as the selection of microorganisms, substrates, supports, and bioreactors for scale‐up, remain significant challenges. A major drawback of SSF is the limited understanding of the physiology of fungi and other microorganisms under these conditions. Additionally, controlling process parameters and scaling up from laboratory to industrial levels pose further difficulties. In SSF systems, scaling up often results in excessive heat generation and system heterogeneity, which can be problematic for heat‐sensitive products or enzymes prone to denaturation. Consequently, the complexity of system design and the need for standardization to ensure cost‐effectiveness and ease of management are among the primary challenges of SSF. Future advancements in SSF are likely to focus on continuous research to optimize scale‐up processes for industrial applications, enabling the sustainable and environmentally friendly production of high‐value products.

## Author Contributions

**Seyyed Mohammad Ali Noori:** formal analysis (equal), project administration (equal), resources (equal), validation (equal). **Mohammad Hojjati:** conceptualization (equal), data curation (equal), supervision (equal), writing – review and editing (equal). **Reihaneh Sorourian:** investigation (equal), methodology (equal), writing – original draft (equal), writing – review and editing (equal).

## Conflicts of Interest

The authors declare no conflicts of interest.

## Data Availability

Data will be made available on request.
